# Clinical utility of decarboxylation prothrombin combined with α-fetoprotein for diagnosing primary hepatocellular carcinoma

**DOI:** 10.1042/BSR20180044

**Published:** 2018-10-05

**Authors:** Jun Fu, Yanyan Li, Zhanzhan Li, Na Li

**Affiliations:** 1 Department of Oncology, Xiangya Hospital, Central South University, Changsha 410008, Hunan Province, China; 2 Department of Outpatient, Xiangya Hospital, Central South University, Changsha 410008, Hunan Province, China

**Keywords:** Alpha-fetoprotein, Decarboxylation prothrombin, Hepatocellular cancer, Meta-analysis

## Abstract

We conducted a comprehensive analysis to evaluate clinical utility of decarboxylation prothrombin combined with α-fetoprotein (AFP) for diagnosing primary hepatocellular carcinoma (HCC). Systematical searches were performed in PubMed, Web of Science, China National Knowledge Internet, and Wangfang databases. The bivariate random-effect model was used to calculate the pooled sensitivity, specificity, positive likelihood ratio (PLR), negative likelihood, diagnostic odds ratio (DOR), and summary area under the curve (AUC). Fourteen studies were included in the meta-analysis. For decarboxylation prothrombin, the overall pooled parameters are as follows: sensitivity: 79% (95% confidence interval (CI): 74–84%), specificity: 91% (95%CI: 87–93%), PLR: 8.42 (95%CI: 5.79–12.23), negative likelihood ratio (NLR): 0.23 (95%CI: 0.17–0.30), DOR: 37.09 (95%CI: 21.37–64.36), summary AUC: 0.92 (95%CI: 0.89–0.94); for combined diagnostic, the overall pooled parameters were as follows: sensitivity: 91% (95%CI: 85–95%), specificity: 83% (95%CI: 74–89%), PLR: 5.26 (95%CI: 3.53–7.83), NLR: 0.11 (95%CI: 0.07–0.18), DOR: 47.14 (95%CI: 30.09–73.85), summary AUC: 0.94 (95%CI: 0.91–0.95). The serum decarboxylation prothrombin showed a relatively higher diagnostic specificity for primary HCC and decarboxylation prothrombin combined with AFP exhibited can improve sensitivity for HCC than any of the biomarkers alone.

## Introduction

The hepatocellular carcinoma (HCC) is the fifth most common cancer in men and seventh in women (17.4 and 6.7 per 100000 persons per year, respectively), and ~750 thousand new cases were diagnosed each year around the world [1]. Seventy percent of all new HCCs worldwide occur in Asia, with patients in the People’s Republic of China accounting for 55% of liver cancer deaths each year [2]. HCC mostly occurs in people with cirrhosis of the liver, and so risk factors generally include factors which cause chronic liver disease that may lead to cirrhosis: chronic viral hepatitis, alcohol abuse, aflatoxin, non-alcoholic steatohepatitis, α1-antitrypsin deficiency, and so on [3]. HCC is the leading cause of cancer-related deaths in some developing countries, with higher degree of malignancy and poor prognosis (almost without exception, those who develop HCC each year die within 12 months), and severely threatens the public health. The data from epidemiological investigations indicate that HCC is a complex and multistage process disease with high incidence, high mortality, highly malignant and invasive, and metastatic [4]. The mortality of HCC was the second to gastric cancer [5]. However, effective treatment with less side effects is scarce [6]. More and more researchers pay more attention on early diagnostic with the expectation of early intervention. Serum biomarkers were still commonly used for tumor diagnostic. Some traditional tumor biomarkers such as α-fetoprotein (AFP) had been widely used in clinical practice. Many studies also reported the diagnostic accuracy of AFP for HCC. The practitioner found that solely AFP still had some false positive (FP) or false negative (FN) rate. It was reported that the AFP level would increase when patients experienced some special disease activities such as active liver injury, some gastrointestinal tumors, and even during pregnancy. Therefore, we further considered the combined diagnostic of serum biomarkers [7]. Des-γ-carboxy-prothrombin (DCP) was a kind of des-γ protein induced by vitamin K absence or antagonist-II (PIVKA-II) and was first suggested to have high sensitivity for HCC in 1984. Up to now, lots of studies have reported that the diagnostic ability of DCP was significantly higher than AFP for HCC, and combined application can further improve the accuracy [8–10]. However, the accurate estimations of DCP and combined diagnostic have not been reported because single study still has some limitations such as population, sample size, and application of different gold standards. We conducted a comprehensive analysis to evaluate clinical utility of decarboxylation prothrombin combined with AFP for diagnosing HCC.

## Materials and methods

### Search strategy

We performed literatures search in PubMed, Web of Science, China National Knowledge Internet, and Wangfang databases from 31 May 1984 to 20 March 2018. We used the following search terms: DCP, protein induced by vitamin K absence or antagonist-II, primary HCC, diagnostic test, DCP, and AFP. Two investigators independently searched the literatures. The search was limited to English and Chinese publications. We also retrieved the references lists of some reviews and articles for potentially included studies. The search strategy was presented in Supplementary material S1.

### Criteria for inclusion and exclusion

The included studies must meet the following criteria: (i) all patients were confirmed by pathologic biopsy and did not receive relevant treatment; (ii) evaluating diagnostic ability of DCP or AFP, or both for HCC; (iii) providing the four-folds data for further analysis (TP, true positive; FP, false positive; TN, true negative; FN). Studies which could not supply enough data, replicates with secondary HCC or received treatment were excluded. Non-diagnostic studies, reviews, comments, case reports, meeting materials, and animal experiments were also excluded. For replicates’ studies, the latest data were used.

### Data extraction and assessment of quality

A standard Excel datasheet was built for information extraction. Two investigators independently conducted the data extraction. For each included study, the following information was extracted: the first author of study, year of publication, source of sample, methods of examination, gold standard, sample size (case/control), total sample size, index, four-folds data (TP, FP, TN, FN), sensitivity, and specificity. We used the updated Quality Assessment of Diagnostic Accuracy Studies 2 (QUADAS-2) to assess the qualities of selected studies. This scale evaluated the quality of study from four domains: patient selection index test, reference standard, and flow of patients. Each domain item was further broken down into several subitems designated as low risk, high risk, or unclear risk according to the study [11].

### Statistical analysis

We used the Stata 14 version (StataCorp LP, College Station, TX, U.S.A.) to conduct all the analyses. Spearman correlation coefficient was used to evaluate the threshold effect [12]. The heterogeneity was assessed via Chi-square and *I^2^* statistic. *P*<0.05 or *I^2^* > 50% means the existence of heterogeneity [13]. A bivariate random-effect model was used to calculate all pooled parameters including sensitivity, specificity, positive likelihood ratios (PLRs), negative likelihood ratios (NLRs), diagnostic odds ratios (DORs), and their 95% confidence interval (CI) [14]. We also estimated the summary receiver operator characteristic curve to evaluate the diagnostic ability. The area under the curve (AUC) reflects the discrimination ability. The AUC ranged from 0 to 1. The diagnostic ability could be useful when AUC > 0.5 or represents a poor test [15,16]. We used linear regression test to evaluate the publication bias [17]. *P*<0.05 was considered as a significant level.

## Results

### Study selection

**Figure 1:**
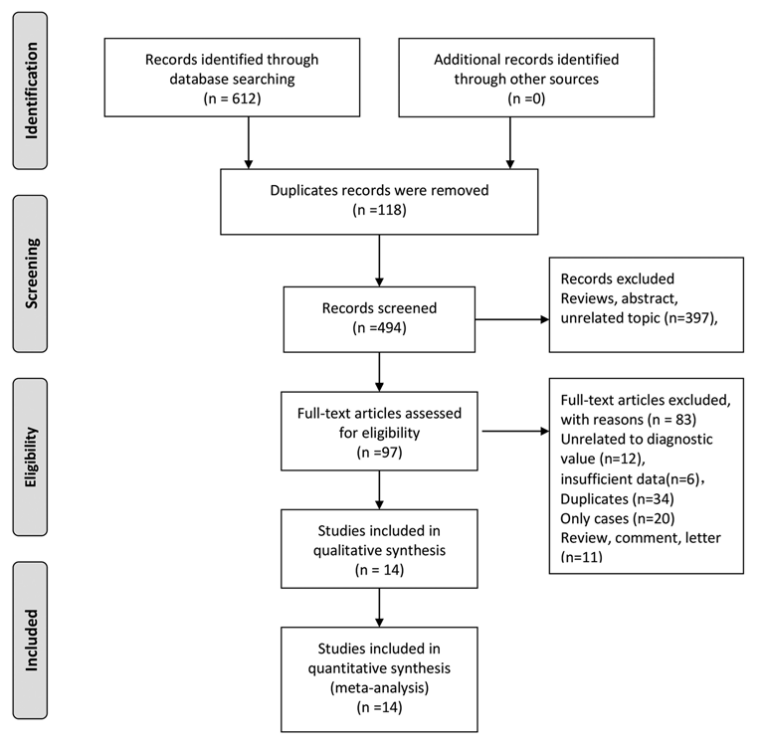
The flow chart of study selection

Two investigators performed literature searches in the online databases. Our initial search returned 612 records and identified zero record through additional methods. One hundred and eighteen records were excluded because of duplicates. Three hundred and ninety-seven records were excluded because of different kinds of reasons such as reviews and unrelated topics. Ninety-seven articles were assessed via full-text articles, and 83 articles were excluded, including 12 unrelated to diagnostic value, 6 records with insufficient data, 34 duplicates, 20 case reports and 11 reviews, comments, and letters. Finally, 14 studies were included in the qualitative and quantitative syntheses [8,16–28]. Figure 1 represented the flow chart of study selection.

### General characteristics and quality of included studies

All included studies were published from 2012 to 2016. Samples of all studies were from serum. All study patients were confirmed by pathologic biopsy. All studies used the enzyme-linked immunosorbent method to detect decarboxylation prothrombin, and most of studies applied ECL immunoassay to detect AFP. The sample size ranged from 126 to 635, with a total number of 8084. Of all the included studies, 14 studies solely evaluated the diagnostic ability of DCP for HCC, and 14 studies used AFP combined with DCP for diagnostic. The sensitivity of single studies ranged from 58 to 97%, and specificity ranged from 56 to 97%. The detailed characteristics were presented in Tables 1 and 2. All the included studies received moderately high scores from the QUADAS-2 quality assessments. The assessment of quality was presented in Supplementary materials S2 and S3.

**Table 1:** General characteristics included in the meta-analysis

Author	Year of publication	Source of sample	Reagent kits and machine		Cancer stage	Methods of examination		Gold standard
			DCP	AFP		DCP	AFP	
**Gao** [18]	2012	Serum	Alisei	Elecsys2010	All stages	ELISA	ECLIA	Pathologic biopsy
**Liu** [19]	2012	Serum	Human DCP kit	Human-L3 kit	All stages	ELISA	ELISA	Pathologic biopsy
**Li** [20]	2013	Serum	–	ABBOTT i2000SR	–	ELISA	ELISA	Pathologic biopsy
**Song** [21]	2014	Serum	ED036, Eissai	–	–	ELISA	ECLIA	Pathologic biopsy
**Pu** [22]	2014	Serum	LUMI-PULSE G1200	Cobase 601	–	ELISA	ECLIA	Pathologic biopsy
**Zhu** [23]	2014	Serum	LUMI-PULSE G1200	Cobase 601	All stages	ELISA	ECLIA	Pathologic biopsy
**Fu** [24]	2015	Serum				ELISA	ECLIA	Pathologic biopsy
**Lin** [8]	2015	Serum	LUMI-PULSE G1200	Cobase 601	All stages	ELISA	ECLIA	Pathologic biopsy
**Yu** [25]	2016	Serum	LUMI-PULSE G1200	AFP Reagent kit, ARTHITECT i2000	All stages	ELISA	ECLIA	Pathologic biopsy
**Shen** [16]	2016	Serum	LUMI-PULSE G1200	–	–	ELISA	ELISA	Pathologic biopsy
**Zheng** [26]	2016	Serum	Sigma RS-232	Sigma-RS-232	All stages	ELISA	ECLIA	Pathologic biopsy
**Huang** [17]	2016	Serum	LUMI-PULSE G1200	Cobase 601	All stages	ELISA	ECLIA	Pathologic biopsy
**Lu** [27]	2016	Serum	LUMI-PULSE G1200	Cobase 601	All stages	ELISA	ECLIA	Pathologic biopsy
**Huang** [28]	2016	Serum	LUMI-PULSE G1200	Cobase 601	All stages	ELISA	ECLIA	Pathologic biopsy

Abbreviation: ECLIA, electrochemiluminescence immunoassay.

**Table 2:** Parameter of included studies in the meta-analysis

Author	Year	Sample size (case/control)	Total	Index	TP	FP	FN	TN	Sensitivity (%)	Specificity (%)
Gao [18]	2012	76/96	173	DCP	57	2	19	94	75	97
Liu [19]	2012	66/60	126	DCP	58	3	8	57	88	95
Li [20]	2013	198/414	612	DCP	139	82	59	332	70	80
Song [21]	2014	550/85	635	DCP	393	10	157	75	71	88
Pu [22]	2014	100/265	365	DCP	74	27	26	238	74	89
Zhu [23]	2014	136/192	328	DCP	115	18	21	174	84	90
Fu [24]	2015	100/100	200	DCP	76	11	24	89	76	88
Lin [8]	2015	100/190	290	DCP	94	21	6	169	94	88
Yu [25]	2016	45/138	183	DCP	26	10	19	128	58	92
Shen [16]	2016	103/156	259	DCP	72	11	31	145	70	92
Zheng [26]	2016	70/150	220	DCP	63	4	7	146	90	97
Huang [17]	2016	100/281	381	DCP	80	20	20	261	80	93
Lu [27]	2016	82/95	177	DCP	59	32	23	63	72	66
Huang [28]	2016	80/188	268	DCP	72	28	8	160	90	85
Liu [19]	2012	66/60	126	AFP + DCP	66	22	0	38	100	63
Li [20]	2013	198/414	612	AFP + DCP	172	97	26	317	87	76
Song [21]	2014	550/85	635	AFP + DCP	456	13	94	72	82	84
Pu [22]	2014	100/265	365	AFP + DCP	81	63	19	202	81	76
Zhu [23]	2014	136/192	328	AFP + DCP	126	20	10	172	92	89
Fu [24]	2015	100/100	200	AFP + DCP	61	1	39	99	61	99
Lin [8]	2015	100/190	290	AFP + DCP	98	39	2	151	98	79
Yu [25]	2016	45/138	183	AFP + DCP	40	20	5	118	88	85
Shen [16]	2016	103/156	259	AFP + DCP	87	18	16	138	84	88
Zheng [26]	2016	70/150	220	AFP + DCP	67	53	3	97	95	64
Huang [17]	2016	100/281	381	AFP + DCP	90	24	10	257	90	91
Huang [28]	2016	80/188	268	AFP + DCP	78	82	2	106	97	56

### Pooled diagnostic accuracy of DCP for HCC

**Figure 2:**
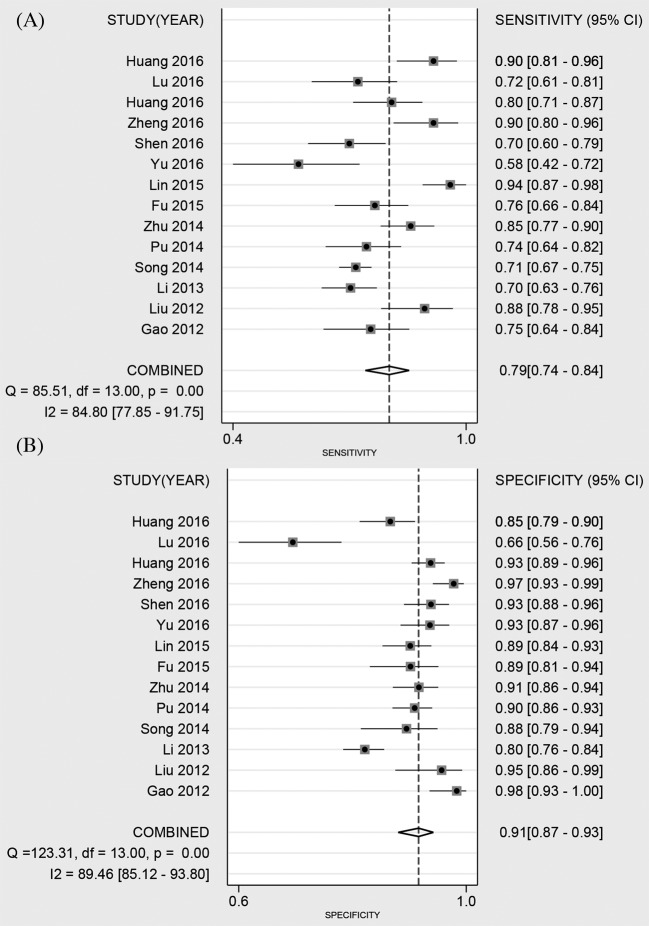
Forest plot of pooled sensitivity (A) and specificity (B) of DCP for primary HCC

Fourteen studies reported the diagnostic ability of DCP for HCC, including 1806 positive subjects and 2410 negative subjects. The Spearman test showed no threshold effect (*r* = −0.134, *P*=0.647). The heterogeneity within studies was high (*I^2^* = 84.80, *P*<0.001), and the random-effect model was used. The pooled sensitivity was 79% (95%CI: 74–84%, Figure 2A), and the specificity was 91% (95%CI: 87–93%, Figure 2B). The pooled PLR and NLR were 8.42 (95%CI: 5.79–12.23) and 0.23 (95%CI: 0.17–0.30), respectively. The DOR was 37.09 (95%CI: 21.37–64.36). The Fagan diagram for evaluating the diagnostic ability of DCP for HCC was shown in Figure 6A. The pre-test probability was ~20%, and the post-test probability was 69% with a PLR of 8. The Figure 4 given the summary receiver operating characteristic (SROC) curve. The pooled AUC was 0.92 (95%CI: 0.89–0.94), which suggested that DCP had a relatively high diagnostic ability for HCC.

### Pooled diagnostic accuracy of DCP combined AFP for HCC

**Figure 3:**
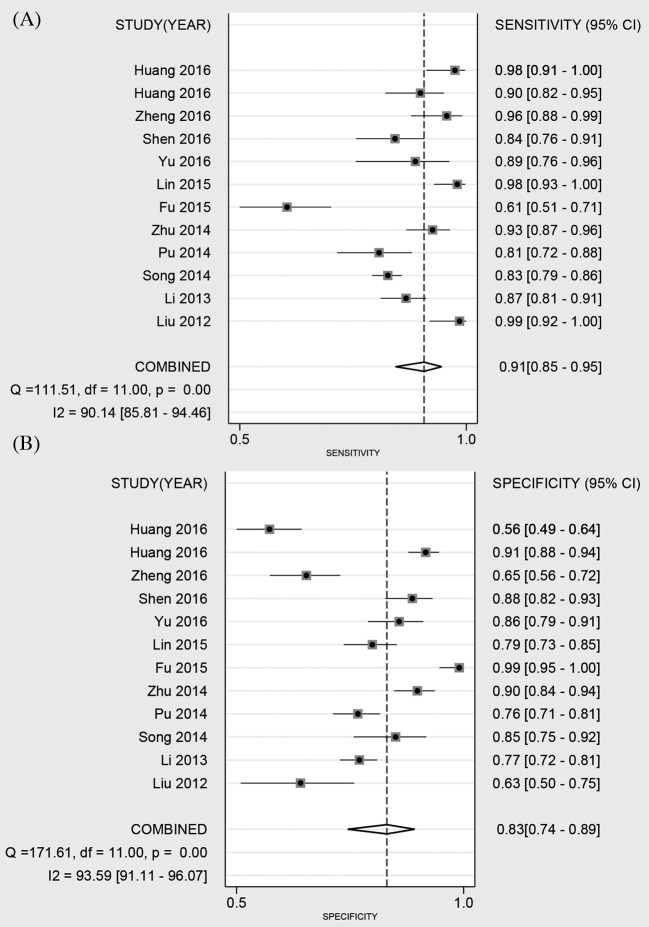
Forest plot of pooled sensitivity (A) and specificity (B) of DCP combined with AFP for primary HCC

Twelve studies reported the diagnostic ability of DCP for HCC, including 1649 positive subjects and 2219 negative subjects. The Spearman test found no threshold effect (*r* = 0.510, *P*=0.089). The heterogeneity within studies was high (*I^2^* = 90.14–93.59%), the random effect model was used. The pooled sensitivity was 91% (95%CI: 85–95%, Figure 3A), and the specificity was 83% (95%CI: 74–89%, Figure 3B). The pooled PLR and NLR were 5.26 (95%CI: 3.53–7.83) and 0.11 (95%CI: 0.07–0.18), respectively. The DOR was 47.14 (95%CI: 30.09–73.85). The Fagan diagram for evaluating the diagnostic ability of DCP for HCC was presented in Figure 6B. The pre-test probability was ~20%, and the post-test probability was 59% with a PLR of 5. Figure 5 gave the SROC curve. The pooled AUC was 0.94 (95%CI: 0.91–0.95), which suggested that DCP combined with AFP had a high diagnostic ability for HCC.

**Figure 4:**
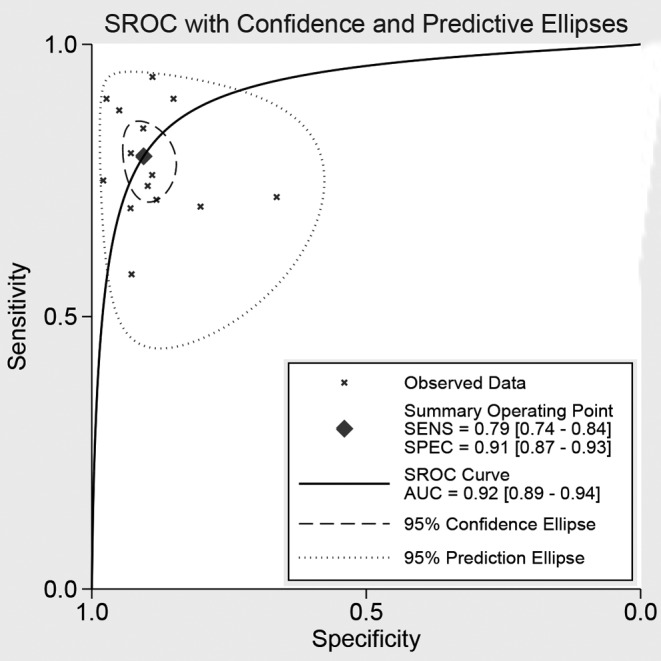
The symmetric ROC curve of DCP for cancer

**Figure 5:**
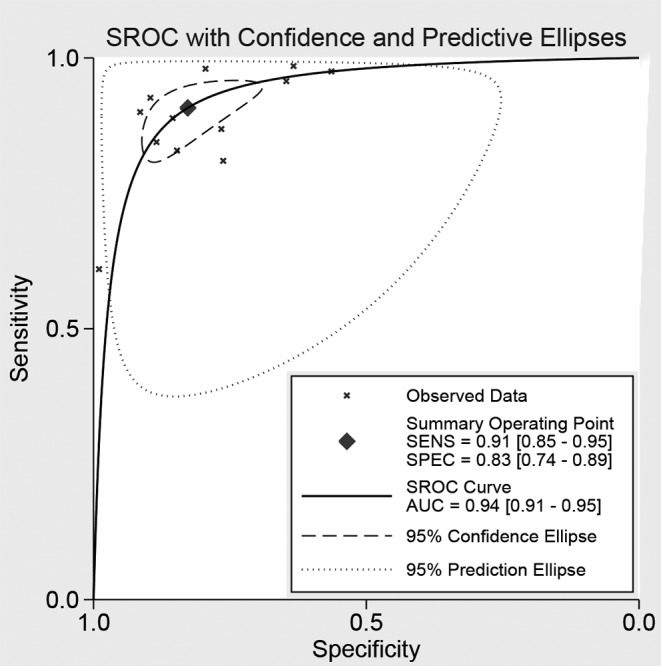
The symmetric ROC curve of DCP combined with AFP for cancer

**Figure 6:**
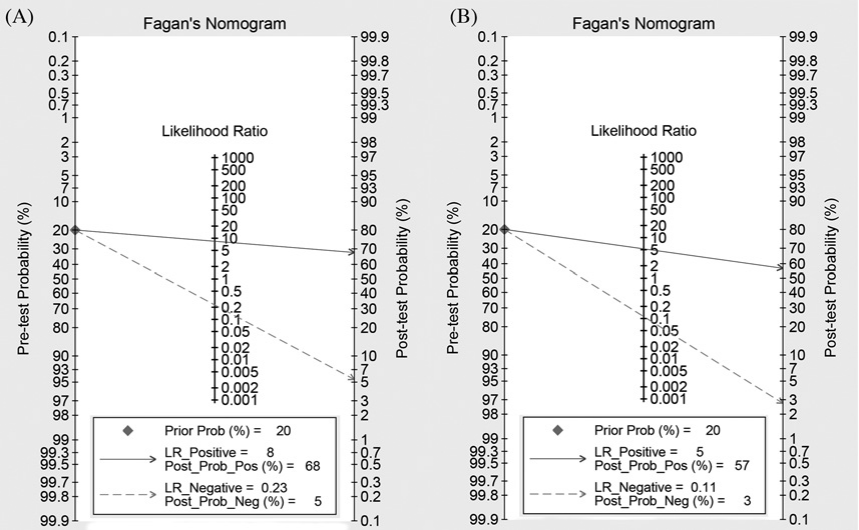
Fagan diagram evaluating the overall diagnostic value of DCP combined with AFP for cancer ((**A**) DCP; (**B**) DCP and AFP).

### Publication bias

**Figure 7:**
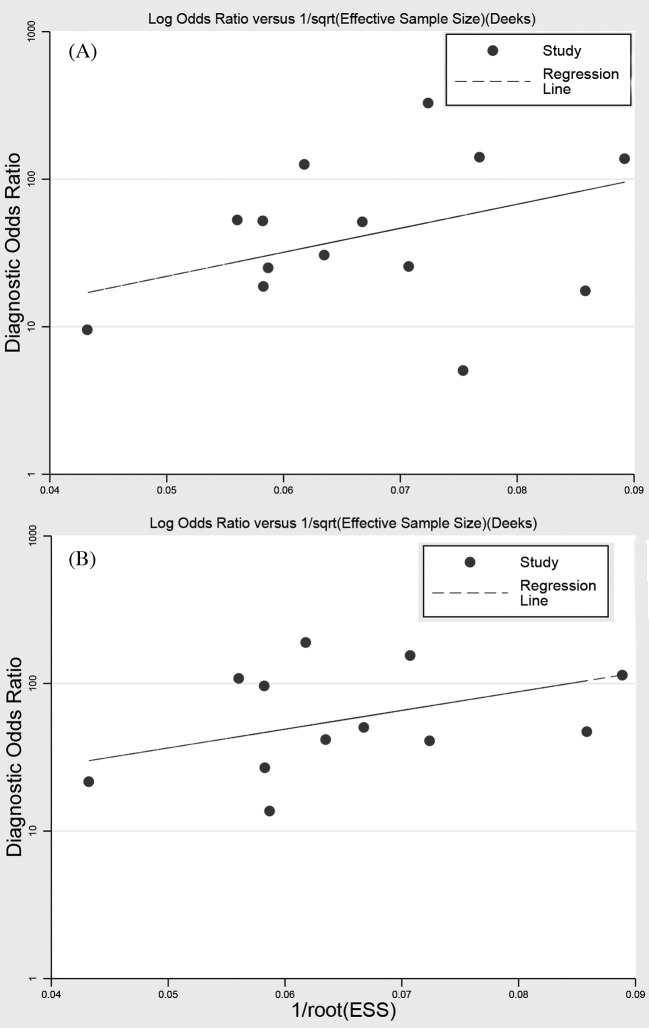
Line regression plot of publication bias ((**A**) DCP; (**B**) DCP, and AFP)

For DCP and DCP combined with AFP, we used the linear regression to evaluate the publication bias. As shown in Figure 7, the *P*-value of slope coefficient of publication bias was 0.133 for DCP, and 0.184 for DCP combined with AFP. No publication bias was found, and the present results were stable.

## Discussion

This is the first meta-analysis to evaluate the clinical utility of decarboxylation prothrombin combined with AFP for diagnosing primary HCC. All studies included in this analysis are diagnostic tests with moderately high quality. The data could give greater power to assess the diagnostic accuracy of AFP and combined DCP for primary HCC. We pooled the sensitivity, specificity, and summary AUC that used DCP and combined AFP for diagnosing HCC through bivariate random effect models and found that both DCP and DCP combined AFP have relatively high diagnostic ability for HCC. The combined diagnostic can provide greater sensitivity, and the DCP have higher specificity.

Single tumor biomarker has some limitations for clinical diagnostic. In the past, AFP was a prior option for diagnosing primary HCC. Previous studies reported AFP could appear at 8–11 months before symptoms [29]. However, the sensitivity of AFP was low for early screening, and 30–35% of patients with primary HCC cannot detect AFP. Besides, patients with chronic hepatitis or liver cirrhosis also have higher level of AFP. It was estimated that 85% patients with HCC were followed by chronic hepatitis [30]. Missed diagnostic can happen easily under these situations. Thrombin is the precursor of the inactive thrombin, which is synthesized by the liver and converted into an activated thrombin form through vitamin K, as the auxiliary factor γ carboxylation. When the liver becomes cancerous, the synthesis of γ carboxylic acid, which is less than normal structure, forms an abnormal prothrombin [26]. The DCP of normal blood serum is very low in content and cannot be detected in general. However, the content of DCP in patients with liver disease increases, which can be detected by enzyme-linked immunoassay [31]. The dickkopf-1 or DCP alone has shown less than satisfactory sensitivity and specificity for the detection of HCC [32].

In the present comprehensive analyses, we found that DCP achieved the overall pooled sensitivity of 79% (95%CI: 74–84%) and specificity of 91% (95%CI: 87–93%). This result suggested that the diagnostic ability of DCP for HCC was moderate, but still lower than combined AFP with sensitivity of 91% (95%CI: 85–95%), specificity of 83% (95%CI: 74–89%). For AUC, the AUC of DCP was 0.92 (95%CI: 0.89–0.94) while the combined AFP was 0.94 (95%CI: 0.91–0.95). Moreover, the single DCP had higher NLR (0.23 compared with 0.11) than combined AFP but lower PLR (37.09 compared with 47.14). Higher positive likelihood meant higher specificity and lower NLR meant lower FN rate. These results indicated that combined AFP has higher diagnostic ability. In fact, many previous studies also showed combined application can improve diagnostic ability of cancer. Lu et al. [33] found that combined detection of plasma *miR-127-3p* and HE4 improves the diagnostic efficacy of breast cancer. Some indictors achieved higher diagnostic ability when combined with other biomarkers. Lubowicka reported that MMP-9 had shown the usefulness in the diagnosis of cervical cancer, but only in the combined analysis with CA 125 [34]. The AUC revealed that DCP had a better accuracy than AFP in diagnosis of HCC (0.891 compared with 0.813, *P*<0.05). The analysis of indicators by logistic regression model indicated that the receiver operator characteristic (ROC) result of joint predictor (DCP, AFP joint detection) was 0.932, superior to the DCP or AFP alone [18]. The combined diagnostic ability had obvious advantage.

The application of serum markers is still important for HCC diagnostic. The AFP alone has some limitations, the AFP level of 30–40% patients with HCC was not significantly elevated while the increased AFP level was found in normal health [35]. Some patients with cirrhosis and/or hepatic inflammation can have an elevated AFP, even without the presence of a tumor. The test had a sensitivity of 39–65%, a specificity of 76–94%, and a positive predictive value of 9–50% for the presence of HCC in previously published studies [36]. Therefore, new serum markers are required. The combined diagnostic ability has obvious advantage. Though the diagnostic meaning of some serum markers of HCC has been downgraded and the importance of imaging had been highlighted, the serum markers still can be used in the clinical diagnostic of HCC in the absence of sensitive imaging methods. For early screening, serum markers and imaging should be considered together.

Our study still has some limitations. First, our study calculated the differences of examination methods, it was reported that diagnostic of DCP from ELSIA was different electrochemiluminescence immunoassay (ECLIA). Second, the control population of some studies may include some patients with hepatitis B infection, this may generate some bias. Third, the structure of population in different studies was different such as age, gender ratio. This could be one of the reasons that caused heterogeneity. Finally, the results showed there was no threshold effect. But the heterogeneity within studies were high, which meant the heterogeneity caused by other sources. The available information was limited, and meta-regression and subgroup analyses cannot be further conducted.

In conclusion, the serum DCP has a relatively higher diagnostic specificity for HCC. The combined diagnostic of AFP and DCP can improve sensitivity for HCC than any of the biomarkers alone. The tests are convenient and inexpensive, and may serve as a valuable addition to current options for the diagnostic of HCC.

## Supporting information

**supplementary Figure:** 

## Abbreviations

AFP: α-fetoprotein
AUC: area under the curve
CI: confidence interval
DCP: des-γ-carboxy-prothrombin
DOR: diagnostic odds ratio
ELISA: enzyme linked immunosorbent assay
FN: false negative
FP: false positive
HCC: hepatocellular carcinoma
HE4: human epididymis protein
MMP-9: matrix metalloprotein-9
NLR: negative likelihood ratio
PIVKA-II: protein induced by Vitamin K absence or antagonist-II
PLR: positive likelihood ratio
QUADAS-2: quality assessment of diagnostic accuracy study 2
ROC: receiver operating characteristic
SROC: summary ROC
TP: true positive
TN: true negative
