# Supplementary material for: Clinical utility of decarboxylation prothrombin combined with α-fetoprotein for diagnosing primary hepatocellular carcinoma
Source: Biosci Rep. 2018 Oct 5;38(5):BSR20180044. doi: 10.1042/BSR20180044 (PMC6172421; doi:10.1042/BSR20180044)
Supplement: Supplementary file 1 [file bsr20180044_Supp1.pdf]

Search Strategy

PubMed (searched on August 10, 2017)

Year (from inception to 2017)

Step 1: “hepatocellular carcinoma”/ all subheading [MeSH] OR “liver cancer”/all subheadings [all fields] OR “liver tumor”/ all subheadings [all fields] OR “HCC” /all subheadings [all fields]

Step 2: “des-γ-carboxy-prothrombin”/all subheadings [all fields] OR “protein induced by vitamin K absence”/all subheadings [all fields] OR “antagonist-Ⅱ” /all subheadings [all fields] OR “DCP” /all subheadings [all fields] OR “AFP” /all subheadings [all fields] AND results from Step 1

Step 3: ‘‘diagnostic test”/all subheadings [ail fields] OR " diagnostic ability”/ all subheadings [all fields] AND results from Step 2

Web of Knowledge (searched on August 10, 2017)

Year (from inception to 2017)

Step 1: “hepatocellular carcinoma”/ [Topic] OR “liver tumor,/ [Topic] OR ‘‘HCC,/ [Topic]

Step 2; " des-γ-carboxy-prothrombin " / [Topic] OR “protein induced by vitamin K absence”/ [Topic] OR “antagonist-Ⅱ” / [Topic] / “DCP” / [Topic] / “AFP” / [Topic] / AND results from Step 1

中文检索策略

中国知网数据库、万方数据库和维普数据库

(检索日期:2017 年 8 月 10 日)

年限(建库至 2017 年)

步骤 1: “原发性肝癌” / [主题词]AND “”

步骤 2:在步骤 1 的基础上“异常凝血酶原” / [关键词]OR “去γ-羧基凝血酶原” / [关键词]

步骤 3:在步骤 2 的基础上加上“DCP” OR “PIVKA-II” / [关键词]

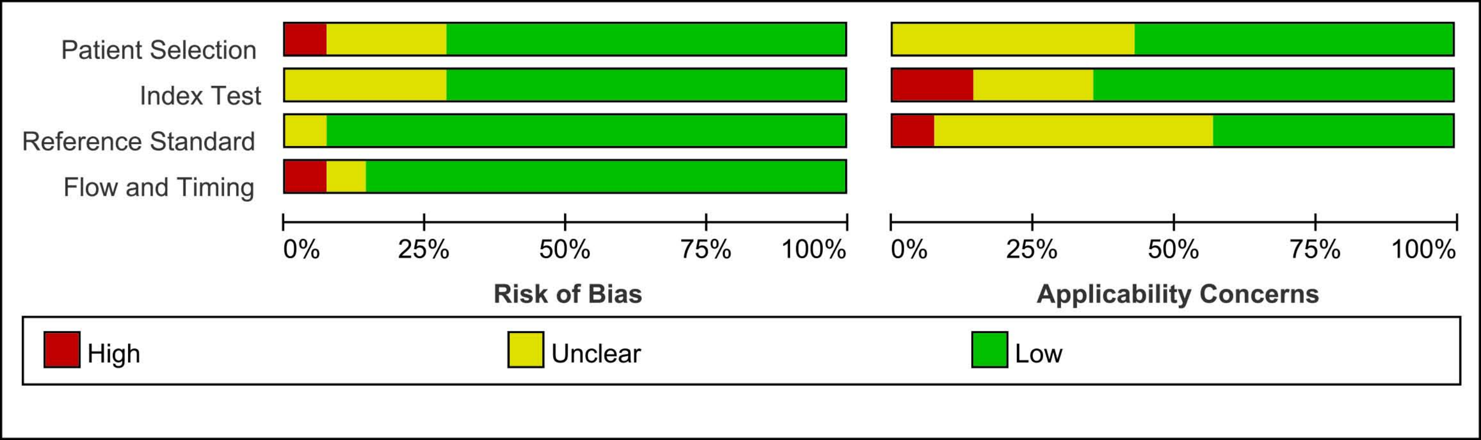
Patient Selection
Index Test
Reference Standard
Flow and Timing
0%
25%
50%
75%
100%
Risk of Bias
Applicability Concerns
High
Unclear
Low

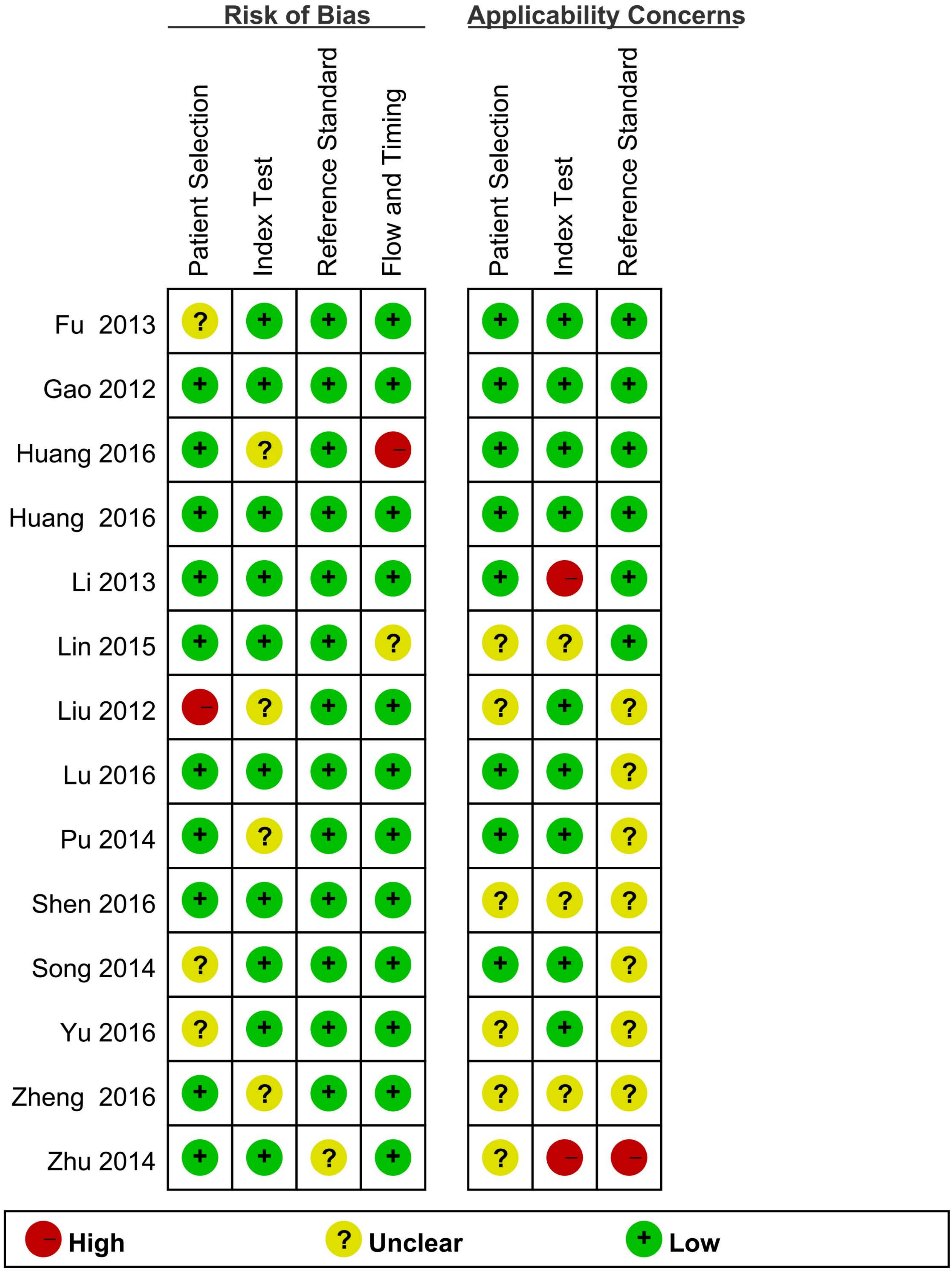
Risk of Bias
Applicability Concerns
Patient Selection
Index Test
Reference Standard
Flow and Timing
Patient Selection
Index Test
Reference Standard
Fu 2013
Gao 2012
Huang 2016
Huang 2016
Li 2013
Lin 2015
Liu 2012
Lu 2016
Pu 2014
Shen 2016
Song 2014
Yu 2016
Zheng 2016
Zhu 2014
High
Unclear
Low
